# Stem cell therapy for COVID-19 pneumonia

**DOI:** 10.1186/s43556-021-00067-8

**Published:** 2022-02-17

**Authors:** Maziar Malekzadeh Kebria, Peiman Brouki Milan, Noshad Peyravian, Jafar Kiani, Soheil Khatibi, Masoud Mozafari

**Affiliations:** 1 Cellular and Molecular Research Centre, Iran University of Medical Sciences, Tehran, Iran; 2 Department of Tissue Engineering and Regenerative Medicine, Faculty of Advanced Technologies in Medicine, Iran University of Medical Sciences, Tehran, Iran; 3 Oncopathology Research Center, Iran University of Medical Sciences, Tehran, Iran; 4 Present Address: Department of Molecular Medicine, Faculty of Advanced Technologies in Medicine, Iran University of Medical Sciences, Tehran, Iran; 5 Babol University of Medical Sciences, Infection Diseases Centre, Mazandaran, Iran

**Keywords:** COVID-19, Coronavirus, Stem cells, Acute respiratory distress syndrome, Tissue regeneration

## Abstract

Severe Acute Respiratory Syndrome Coronavirus 2 (SARS-CoV-2) virus is a highly contagious microorganism, and despite substantial investigation, no progress has been achieved in treating post-COVID complications. However, the virus has made various mutations and has spread around the world. Researchers have tried different treatments to reduce the side effects of the COVID-19 symptoms. One of the most common and effective treatments now used is steroid therapy to reduce the complications of this disease. Long-term steroid therapy for chronic inflammation following COVID-19 is harmful and increases the risk of secondary infection, and effective treatment remains challenging owing to fibrosis and severe inflammation and infection. Sometimes our immune system can severely damage ourselves in disease. In the past, many researchers have conducted various studies on the immunomodulatory properties of stem cells. This property of stem cells led them to modulate the immune system of autoimmune diseases like diabetes, multiple sclerosis, and Parkinson's. Because of their immunomodulatory properties, stem cell-based therapy employing mesenchymal or hematopoietic stem cells may be a viable alternative treatment option in some patients. By priming the immune system and providing cytokines, chemokines, and growth factors, stem cells can be employed to build a long-term regenerative and protective response. This review addresses the latest trends and rapid progress in stem cell treatment for Acute Respiratory Distress Syndrome (ARDS) following COVID-19.

## Introduction

The coronavirus disease 2019 (COVID-19) pandemic virus spread rapidly and led to death from pneumonia. At first, it was thought to be seasonal influenza, but after much research, it became clear that it was a new case. After laboratory research, the type of virus was quickly identified [1]. Coronavirus family member SARS-CoV-2 was the name given to the virus, which spreads via respiratory droplets and contact surfaces [2]. The average incubation period is 3 to 5 days but ranges from 2 to 14 days [3]. However, the World Health Organization (WHO) announced 1 to 11 days recently. Patients with a mild respiratory infection are usually diagnosed with fever and cough, which are the most frequent signs of illness [4]. Among patients, only 14% had acute respiratory symptoms or ARDS [5]. However, the virus has spread around the world and has shown significant mortality. Various drugs are being tested to treat or control this disease. For example, Antiviral drugs, including redeliver and lopinavir-ritonavir, are under investigation, but the effects need further study [6]. Recently, countries have been using the hydroxychloroquine drug. It is used to treat malaria. Although this drug has shown appropriate cure effects, the use of these drugs in the treatment protocol needs to be more investigated. In addition to using these drugs, Chinese researchers have suggested using mesenchymal stem cells (MSCs) [7]. Due to their capacity to self-renew and specialize in functional cell types, mesenchymal stem cells (MSCs) have become an important source of cells for cell-based therapies [8–10]. Studies have recently reported that MSCs modulate the immune system. Researchers also show that inflammatory storms caused by the virus in the lungs may reason for severe pathogenesis [8, 11, 12]. Therefore, MSCs with their modulatory and anti-inflammatory properties, especially interleukin inhibition, can play an essential role in reducing the effects of COVID-19 in the lungs. We focused on the biochemical and clinical effects of mesenchymal cells on COVID-19 in this review research.

## The emergence of COVID-19

**Fig. 1:**
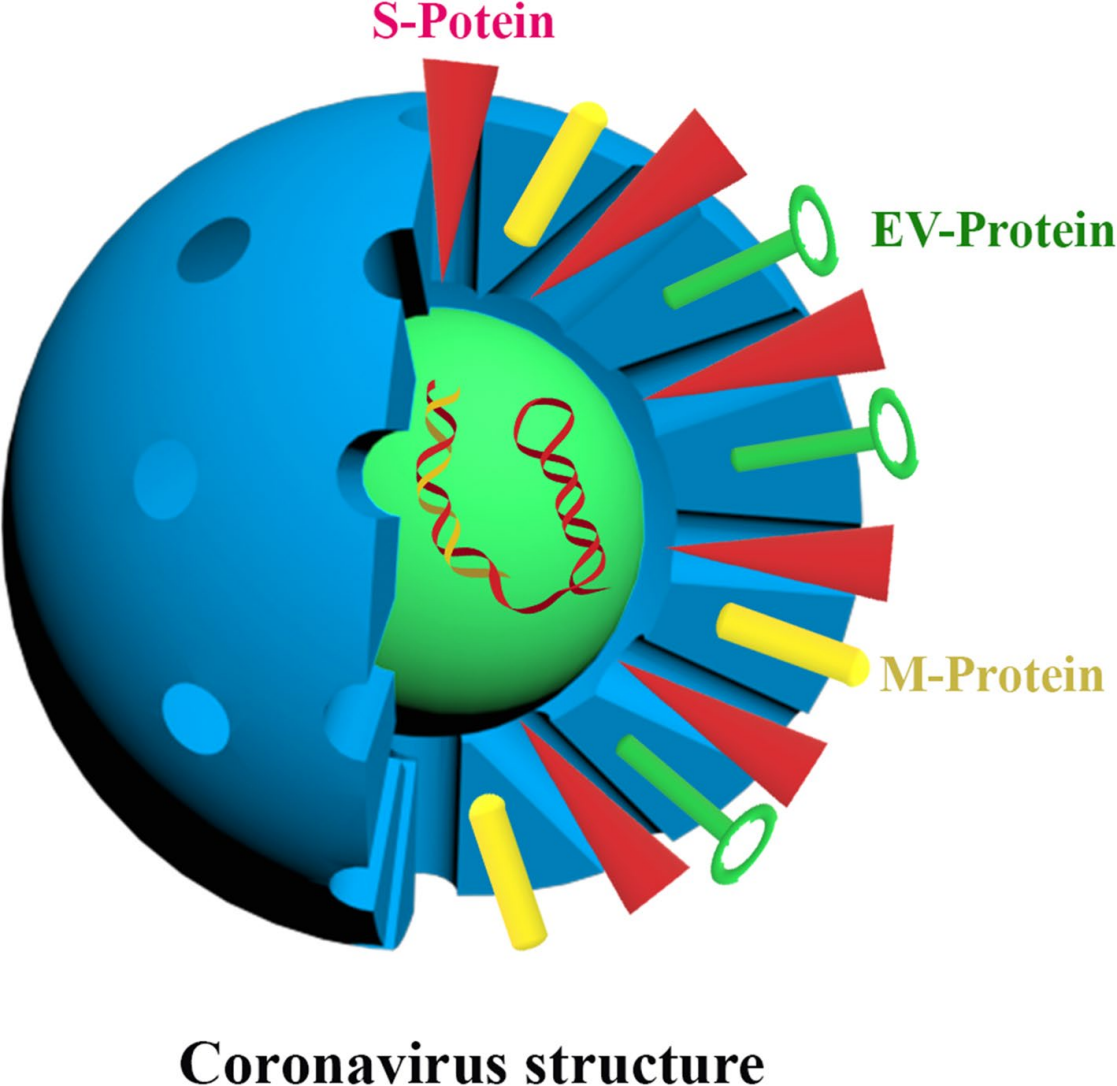
Coronavirus structure. In this structure, surface proteins (The spike protein (S-Protein), Envelope Protein (EV-Protein) and Membrane Protein (M-Protein), nucleotides and crowns of this virus are shown. The corona crown gives this type of virus a unique structure that distinguishes it from other viruses

For convenience, the virus was briefly called the SARS-COV-2 virus, and the WHO assigned the designation COVID-19 to the SARS-COV-2 virus-associated sickness [13, 14]. All animals, including humans, can be infected by single-stranded RNA viruses [15–18]. They were initially identified in 1966 by Tyrell and Bynoe, who grew the viruses from individuals who had the flu. They were known as coronaviruses because of their spherical shape, which had a core-shell and a crown-like top (Fig. 1) [19]. There are four subfamilies of coronaviruses: alpha, beta, gamma, and delta. It is not unusual for gamma and delta-viruses to arise in pigs or birds. Of course, it is important to note that their genome size varies from 26 to 32 KB [1, 20, 21]. The beta virus is the most dangerous and deadly among the groups that can infect humans, while the alpha subgroup has shown milder symptoms in mammals, especially humans. SARS-COV-2 is owned by the B lineage of the beta-coronaviruses [4, 20]. SARS-COV-2 may have been transferred to people by eating marine creatures [22]. However, the identification of the causative agent of this virus has not been entirely determined [23–25]. An important symptom that confirmed this disease was pneumonia [26–28]. Observations show that SARS-COV-2 average incubation time is 3 to 5 days. However, the incubation period of the virus has not been assessed definitely [29, 30].

In most cases, symptoms of the condition begin to emerge within a few days [31]. Cough, fever, exhaustion, and upper respiratory tract infections are among the most common symptoms of coronavirus. About 70% of individuals will get dyspnea and other serious chest symptoms that communicate with pneumonia as a result of the infection [31, 32]. Pneumonia usually begins two to three weeks after the onset of symptoms. Eminent indications of pneumonia consist of decreased oxygen saturation, and blood gas aberration changes evident through chest X-rays, maculate consolidation, alveolar exudation and interlobular contention [33, 34]. The pathophysiology of COVID-19 is similar to SARS-COV-2, which involves an intense inflammatory reaction in the airway resulting in severe lung damage. Accordingly, illness severity can be determined by the host’s immunological response [35, 36]. A cytokine storm, which occurs when the immune system releases large amounts of cytokines in response to a viral infection or secondary infections, can result in 30% of fatal cases. Case numbers: COVID-19 [37, 38]. In these cases, an uncontrolled inflammatory response causes damage to the liver, heart and kidneys. According to, many patients died due to the loss of function of these organs; however, most deaths are caused by lung damage [39, 40].

## Mesenchymal stem cells and therapeutic effects

Embryonic stem cells and adult stem cells are the two primary types of stem cells, distinguished by their origin [41, 42]. Adult stem cells such as mesenchymal stem cells are extremely important [43]. MSCs have the ability to transform into different cell lineage in vitro. Accessible sources and easy proliferation of these cells and autologous make them suitable candidates for cell therapy [44, 45]. A wide range of disorders has been treated with MSCs in the recent decade [46]. The clinical evidence showed that more than two thousand patients received MSCs as autologous and allogenic cells cultured in different diseases and syndromes [47, 48]. By secreting tissue healing factors, they can change into all three germ layers and self-renew. Anti-inflammatory cytokines, vesicles and extracellular vesicles promote tissue and organ regeneration [49–51].

MSCs migrate toward damaged sites by secreting many factors and mechanisms, containing chemokine factors such as CXCR4 binds to its ligand CXCL12 caused trigger cell migration [52, 53]. Tumour necrosis factor (TNFa), interleukin 10, and interferon-gamma (INFg) are all secreted by MSCs after being stimulated by inflammatory stimuli [54, 55]. The connection of MSCs with a damaged microenvironment causes the release of complex factors in the proliferation and differentiation of local progenitor cells [56, 57].

MSCs have the right to control the growth and function of immune cells through their characteristics. For example, inhibiting the generation of antibodies by B cells, repressing NK cell proliferation, decreasing TNF- and IL-12 production, inhibiting the differentiation of monocytes into dendritic cells, etc. . [58–60]. Anti-inflammatory cytokines such as IL-10, on the other hand, increase the number of monocytes, which suggests that these cells can block dendritic cells from causing inflammation [61, 62]. As an additional advantage, MSCs modulate the innate immune system by inhibiting the activity of natural killer cells [63]. By lowering the expression of NKP44, NKG2D receptors and NKP30, MSCs focus on their inhibitory activity of NK cells [64, 65]. MSCs inhibit the production of hydrogen peroxide by activated neutrophils [66]. Therefore, these cells can reduce the intensity of inflammatory stimuli [67]. Immune system reactions can be lowered by using MSCs, according to these research [68, 69]. It is critical to understand how MSCs affect the activity of T lymphocytes since these cells are regarded as the essential cells in the cellular immune system [70, 71]. It has been shown in several research investigations that monoclonal or allogeneic cells or particular antigens suppress the proliferation of T cells in the presence of MSCs [72]. These cells exert their inhibitory function on lymphocyte proliferation by stopping these cells in the G cell cycle. CD4 ^+^ T lymphocytes [T helper, Th] are the main subcategories, each of which has phenotypic characteristics, and It is a self-contained function. Th1 and Th17 an inflammatory subtypes among these subcategories and guide inflammatory pathways [73–75]. The subcategories Th2 and Treg are also known as the population of suppressive cells or regulators of the immune system [76, 77].

From another perspective, MSCs have been caused slight immunogenic potential in vitro and in vivo due to their limited expression of MHC I molecules, the absence of MHC II statement and costimulatory molecules [78].

As a result of the immunomodulatory action of MSCs and their low MHC class I expression, allogeneic stem cell transplantation patients can avoid graft-versus-host disease (GVHD) [79]. Numerous studies have been accomplished about various lineages of MSCs in diseases. For example, mesenchymal cells have been used to treat chronic heart injury, pancreatitis, diabetes, and various viral diseases [80]. Moreover, the role of translation MSC in cardiology, neurology and orthopaedics explain in a few reports, and it is one of the most promising therapeutic cells in these diseases and has also been widely used as a potential therapeutic target [47].

**Fig. 2:**
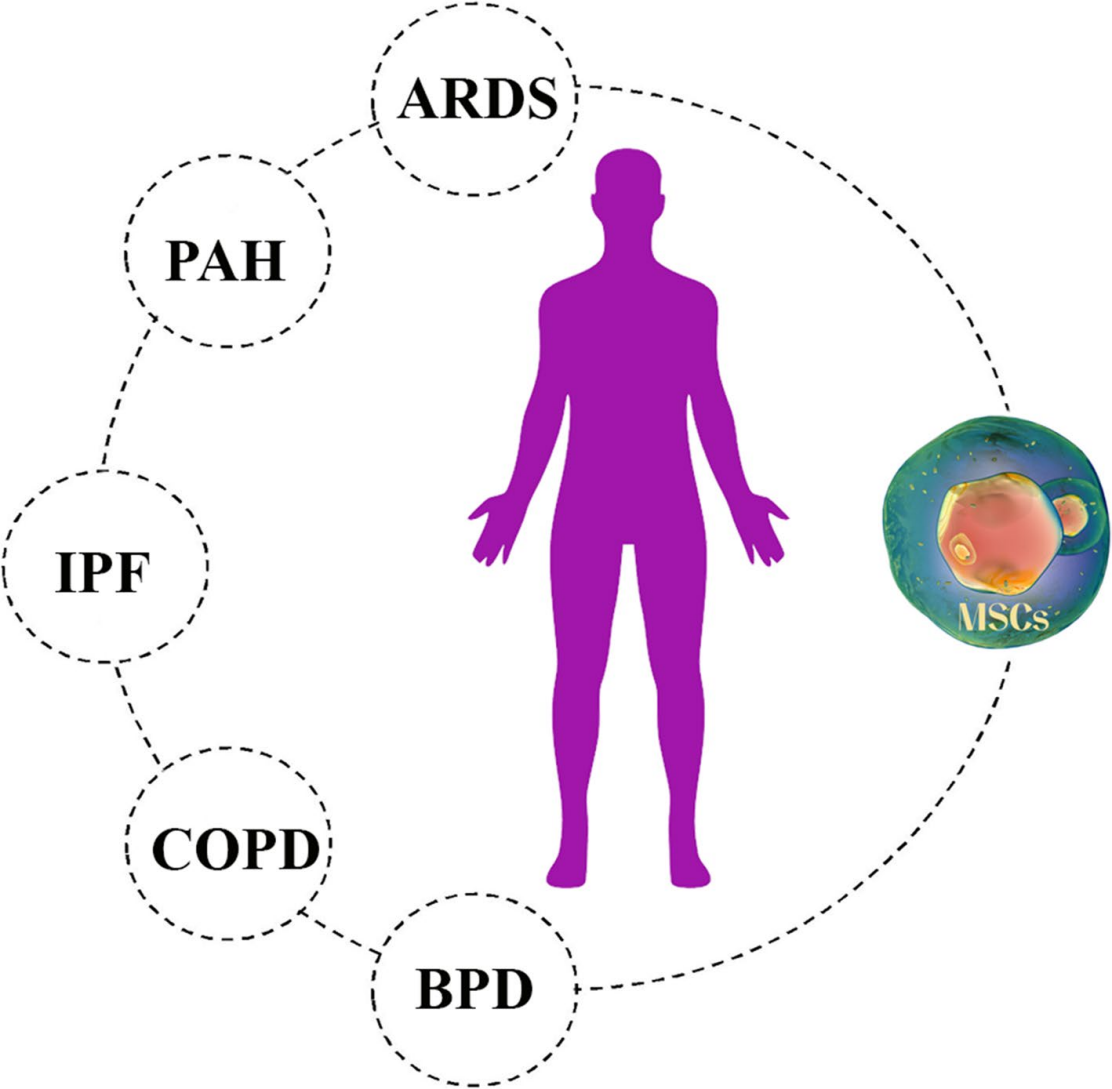
The effect of Mesenchymal stem cells (MSCs) on a variety of lung diseases. These cells have the ability to regenerate damaged alveoli and fibrosis due to their regenerative and immunomodulatory properties. COPD (Chronic obstructive pulmonary disease), PAH (Pulmonary arterial hypertension), ARDS (Acute respiratory distress syndrome), BPD (Bronchopulmonary dysplasia) and IPF (Idiopathic pulmonary fibrosis)

As mentioned, MSCs can be used in viral diseases. Various studies have been shown that damage to MSCs increases the entry of viruses and their pathogenicity. This cell type’s function in HIV treatment, chronic HBV treatment, and influenza virus acute lung injury treatment (ALI) has been examined [81]. Taking the HIV-1 virus as an example, the virus is characterized by the total depletion of newly formed CD4^+^T cells, resulting in severe immunodeficiency in the clinical environment. The most important function of MSCs is to aid in the restoration of the host immune system by decreasing the activation of CD8^+^T cells and enhancing the effectiveness of CD4^+^T cell restitution. On the other hand, HBV is the most common pathogenesis in which 0.5 million people die from HBV-related liver disease or hepatocellular carcinoma worldwide each year [82]. Patients with end-stage liver disease who received human bone marrow-MSC had improved liver function. In acute lung injury (ALI) in influenza, the MSCs mechanism for modulation in the immune system has been used for treatment. Since these cells have been used to treat viral diseases (Fig. 2), researchers have found essential results in the treatment of COVID-19 due to its modulation effect [58].

### The effects of MSCs on acute respiratory distress syndrome [SARS-COV-2]

An infectious agent causes respiratory illnesses. Similar respiratory symptoms can be found in a wide range of viruses, from the common cold to the most severe acute respiratory syndrome [83]. There is currently no authorized medicine or vaccination for COVID-19 illness. However medical organizations and scientists are working to develop a cure. Using emapticap, natalizumab, efalizumab, and convalescent plasma as immunomodulatory and immunoglobulin drugs in treating severe COVID-19 patients is effective [84–87].

**Fig. 3:**
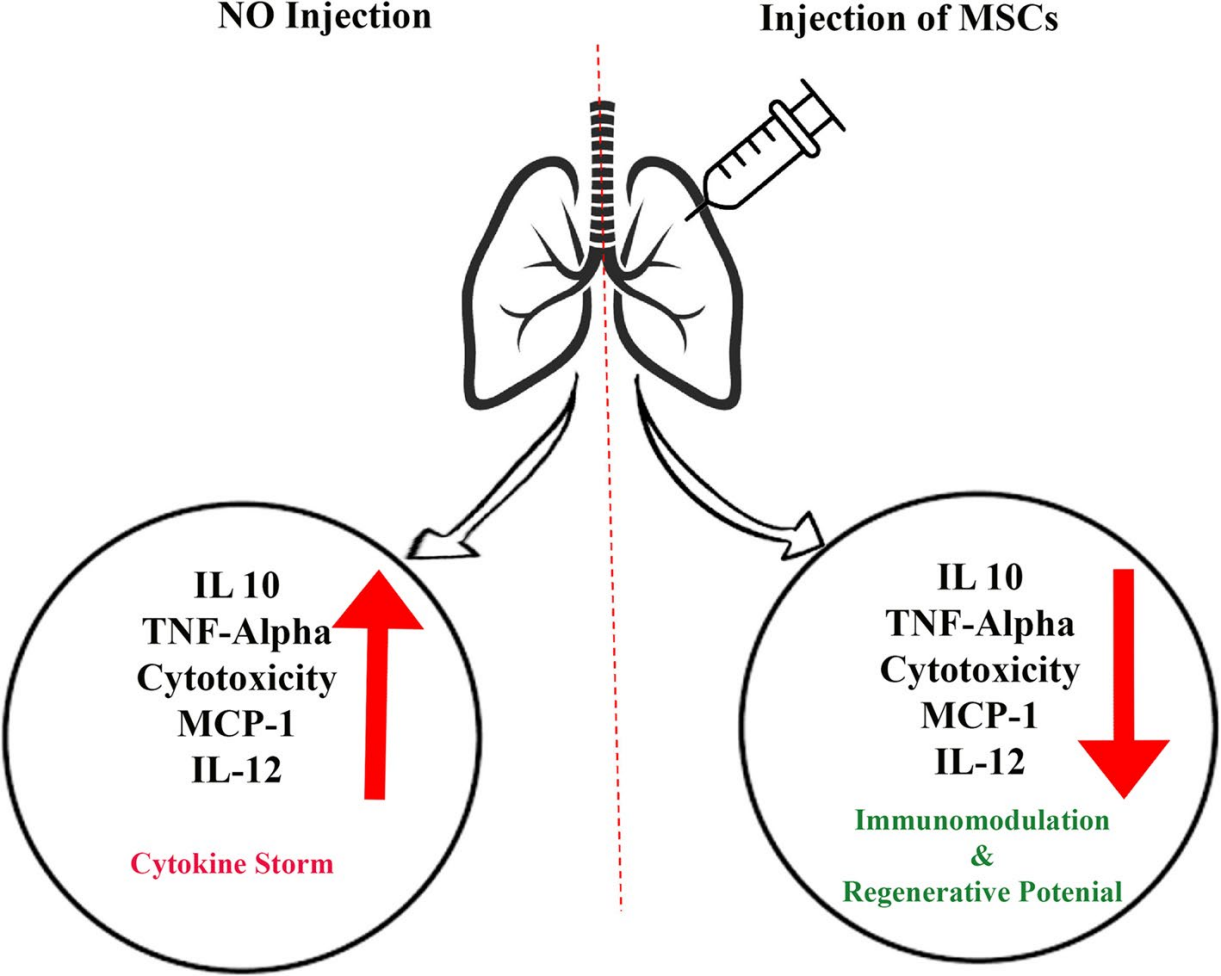
Effects of Mesenchymal stem cells (MSCs) injection on the patient’s lung. Injection of this cell reduces the secretion of interleukins 10, 12 and MCP-1. Reducing the secretion of these inflammatory factors prevents lung and respiratory diseases such as COVID-19

SARS-inflammatory Cov-2’s response is the primary method for destroying the virus, but this activity damages and dysfunctions the body’s tissues. Viral entrance into tissue results in the release of pro-inflammatory molecules such as IL-1, IL-17, TNF-, and INF-. They can regenerate and regulate the immune system, which is useful for cell therapy. Mesenchymal stem cells (MSCs) come from many organs. It has been established that mesenchymal stem cells have considerable angiogenic and healing, anti-apoptotic, and immune-regulating capacities (Fig. 3). In addition, due to the low expression of MHC-I, MHC-II and excitatory molecules, they can generally be characterized as evaporative and immune when used in allogeneic settings. Mesenchymal stem cells modulate the immune system by cell-paracrine-dependent mechanisms, including releasing TNF, IL-10, indolamine 2,3-dioxygenase, adenosine, and extracellular vesicles. Additionally, these mechanisms result in decreased differentiation and activation of other immune cells. When it comes to treating inflammatory illnesses, mesenchymal stem cells are an excellent option. Considering inflammatory diseases, the most consistent data are related to mesenchymal stem cells in transplant therapy. When transplantation takes place, the immunomodulatory effects of mesenchymal cells are already prominent. Following an intravenous injection of cells, mesenchymal stem cell treatment is commonly used. Mesenchymal stem cells (MSCs) were shown to be swiftly struck in the lungs following intravenous injection, and damaged regions increased MSC migration, which is interesting. As a result of lung damage, angiotensin II synthesis in the capillaries increases, resulting in angiotensin II receptor interactions that stimulate MSC migration in-vivo. Anti-inflammatory cytokines and antimicrobial peptides are released by mesenchymal stem cells when they are stuck in the lungs, as described elsewhere in the body. Vaccines, antibodies, antivirals, and RNA-based medications and living therapies utilizing promising cell types like natural killer (NK) cells and stem cells have all been proposed as potential treatments [88, 89]. Therefore, the clinical experience chiefly comprised of antibacterial and antiviral drug treatments have been recognized to prohibit the ingress of these viruses in cell culture or animal models. Furthermore, antiviral drugs are the primary line treatment for COVID-19 induced pneumonia and can effectively obliterate the virus. Antiviral drugs cannot amendment damaged lung cells [10, 90]. Newly, stem cell therapy has become an excellent approved tool for treating viral lung damage. Because attempts to treat lung damage with a variety of drugs have not been successful, the use of cell therapy has been suggested. MSCs have a high ability to repair and regenerate. Due to these properties and modulating the immune system, researchers have considered these cells [91]. Plenty of clinical trials are done for this cell (Table 1).

**Table 1:** Clinical studies of MSCs treatment for patients with COVID-19

Line	^a^NCT Number	Sponsor	Date of registration First/Last	Target Sample Size	Intervention/treatment	disease
1	NCT04252118	Beijing 302 Hospital, China	27/01/2020	20 Male/female 18–70 years old	3 times intravenous injections of 3.0 × 10^7^ MSCs on days 0, 3 and 6 in phase 1	COVID-19
2	NCT04273646	Wuhan Union Hospital, China	14/02/2020	48 Male/female 18–65 years old	0.5 × 10^6^ UC-MSCs/kg body weight intravenously at Day 1, Day 3, Day 5, and Day 7 of the trial period. - Phase 1: -	COVID-19
3	NCT04339660	Puren Hospital Affiliated to Wuhan University, China	01/02/2020 30/06/2020	30 Male or female, 18–75 years old	1 × 10^6^ UC-MSCs /kg body weight suspended in 100 mL saline Phase 1/Phase 2	ARDS
4	NCT04288102	Beijing 302 Hospital, China	05/01/2020 09/07/2020	100 Male/female 18–75 years old	3 times intravenous injections of 4.0 × 10^7^ MSCs on days 0, 3 and 6 in Phase 2	COVID-19
5	NCT04276987	Ruijin Hospital, China	16/02/2020 31/07/2020	30 Male/female 18–75 years old	MSC-derived exosomes [2.0 × 10^8^ nano vesicles/3 ml at Day 1, Day 2, Day 3, Day 4, and Day 5] were inhaled five times a day for five days. Phase One	COVID-19
6	NCT04299152	Tianhe Stem Cell Biotechnologies Inc., China	10/04/2020 10/11/2020	20 Male/female 18–60 years old	Combination Product: Cord Blood Stem Cells [CB-SCs] Educator-Treated Mononuclear Cells Apheresis Phase 2	COVID-19
7	NCT04302519	CAR-T [Shanghai] Biotechnology Co, China	05/03/2020 30/07/2021	24 Male/female 18–75 years old	Use of dental pulp MSCs According to clinical standards, intravenous injection of dental MSCs was performed on days 1, 3 and 7. Phase 1	COVID-19
8	NCT04269525	Tuohua Biological Technology Co. Ltd., China	07/02/2020 30/12/2020	16 Male/female 18–80 years old	UC-MSCs 3.3 × 10^7^ cell number as much as 50 ml in 3 bags [any time] will be infused intravenously on the 1st, 3rd, 5th, and 7th days in Phase 2	COVID-19
9	NCT04371393	Icahn School of Medicine at Mount Sinai, New York City	30/04/2020 30/04/2022	300 Male/female 18 Years and older	2 × 10^6^ MSC It was injected based on body weight and standard of care versus placebo in Phase 3	COVID-19
10	NCT04444271	National Institute of Blood and Marrow Transplant [NIBMT], Pakistan	01/05/2020 30/09/2020	20 Male or female Aged ≥10 years	Frozen MSCs is suspended in 100 ml normal saline solution for urgent use. The injection was given intravenously. Each patient in Experimental will be given 2 × 10^6^ cells/kg MSCs on day 1 to 7 that will be managed all steps. This experiment is in phase 2.	ARDS
11	NCT04392778	Istanbul Bakirkoy DR. SADI Konuk Training and Research Hospital	01/04/2020 30/09/2020	30 Male or female 40–60 years old	Patients who are connected to a ventilator and receive MSCs intravenously. 3 million cells/kg in 0, 3 and 6th day	ARDS
12	NCT04315987	Hospital Vera Cruz, Brasil	01/06/2020 30/08/2020	90 Male or female, Aged ≥18 years	2 × 10^7^ NestaCell® will be enforced intravenously on days 1, 3, 5 and 7 in Phase 2	COVID-19
13	NCT04355728	University of Miami, USA	25/04/2020 31/12/2020	24 Male or female, Patients >/= 18 years old	Heparin and MSCs from the umbilical cord are the best therapy strategies. At 100 × 10^6^ cells/infusion, UC-MSC will be administered intravenously. for Phase 1/Phase 2 has 1 first infusion: 24 h, second Infusion: 72 h	COVID-19
14	NCT04416139	Salvador Zubirán National Institute of Health Sciences and Nutrition Mexico	01/05/2020 01/05/2021	10 Male or female, 18 Years and older	I.V. infusion of MSCs derived from peripheral blood mononucleotides. A single injection of 1 million cells/kg. Phase 2 [Phase 1]	COVID-19
15	NCT04466098	Masonic Cancer Center, University of Minnesota USA	30/07/2020 01/12/2021	30 Male or female, Age 18–80 years	Total volume of 60 mL of thawed product includes MSCs [300 × 10^6^] in DMSO and suspended with Dextran 40 and 5% human serum albumin.Phase 2	COVID-19
16	NCT03818854	University of California, USA	01/01/2020 20/08/2020	120 Male or female, 18 Years and older	10 million cells/kg PBW [predicted body weight] in a single dosage 60–80 min after intravenous administration of allogeneic bone marrow-derived human mesenchymal stromal cells. Phase 2	COVID-19
17	NCT04390139	Banc de Sang i Teixits, Spain	13/05/2020 03/12/2020	30 Male or female, 18 Years to 75 Years	Wharton-Jelly mesenchymal stromal cells on Day 1 and Day 3 [XCEL-UMC-BETA] Each dose of MSC-WJ will consist of the intravenous administration of 1E6cells/Kg Phase 1/Phase 2	COVID-19
18	NCT04333368	Assistance Publique - Hôpitaux de Paris	06/04/2020 06/04/2022	40 Male or female patient, age > 18 years	Human MSC umbilical cord derived from Wharton jelly [1 ml per kg] delivered intravenously within 1 h using a 200 μm filter tube in 150 ml cells on days 1, 3, 5 in Phase 1and Phase 2.	COVID-19
19	NCT04366063	Royan Institute, Iran	05/04/2020 30/12/2020	60 Male or female, 18 Years to 75 Years	**Cell therapy protocol 1** 20 Patients will receive two doses of MSCs 100 × 10^6^ [±10%] at Day 0 and Day 2 plus Conventional treatment **Cell therapy protocol 2** Patients will receive two doses of MSCs 100 × 10^6^ [±10%]at Day 0 and Day 2, intravenously plus two doses of EVs at Day 4 and Day 6 plus conventional treatment Phase 2/Phase 3	ARDS
20	NCT04397796	St. Francis Medical Center Lynwood, California, United States	21/05/2020 27/05/2021	45 18 Years to 80 Years [Adult, Older Adult]	BM-Allo.MSC for Infusion, is manufactured from normal donor derived bone marrow product and are phenotypically CD73+, CD90+, CD105+, and negative for CD14-, CD34-, CD45-, HLA-DR- Phase 1	COVID-19
21	NCT04416139	Instituto Nacional de Ciencias Médicas y Nutrición Salvador Zubirán Mexico City, Mexico	04/06/2020 01/05/2021	10 18 Years and older [Adult, Older Adult]	Infusion IV of Mesenchymal Stem cells Mesenchymal Stem cells from bank will be applied IV, at dose 1 million xKg in a single dose Phase 2	COVID-19
22	NCT04456439	Mesoblast International Sàrl	02/07/2020 18/02/2021	50 2 Months to 17 Years [Child]	Multisystem Inflammatory Syndrome in Children [MIS-C] Involved with Coronavirus Disease [COVID-19] Associated with Remestemcel-L, Human Mesenchymal Stromal Cells. Phase 1	COVID-19
23	NCT04527224	Nature Cell Co. Ltd.	26/08/2020 01/04/2023	10 19 Years to 80 Years [Adult, Older Adult]	Drug: AstroStem-V Allogenic adipose tissue-derived mesenchymal stem cells [AdMSCs] Phase 1/Phase 2	COVID-19
24	NCT04537351	Saint Albans, Victoria, Australia Cynata Therapeutics Limited	03/09/2020 20/05/2021	24 18 Years and older [Adult, Older Adult]	Cymerus mesenchymal stem cells [MSCs], the active ingredient in CYP-001, are produced from patented induced pluripotent stem cells [iPSC] and mesenchyme-angioblast [MCA] manufacturing processes. Two million Cymerus MSCs/kg of body weight (up to a maximum of 200 million cells) will be infused into each participant randomized to CYP-001 on D1 and D3. Phase 1/Phase 2	COVID-19
25	NCT04565665	M D Anderson Cancer Center, Houston, Texas, United States	25/09/2020 30/04/2021	70 18 Years and older [Adult, Older Adult]	Mesenchymal Stem Cell, Given IV Mesenchymal Progenitor Cell, Patients receive MSCs IV over 1–2 h on day 1. Patients may receive a second infusion of MSCs within 7 days after the first Infusion per physician discretion Phase 1/Phase 2	COVID-19
26	NCT04611256	Hospital Regional Lic Adolfo Lopez Mateos, Mexico City	02/11/2020 12/11/2020	20 18 Years to 65 Years [Adult, Older Adult]	Two intravenous Infusion of 1 × 10^6^ adipose tissue derived-MSCs /kg body weight reach on the day 1 [D1] and the day 3 [D3] of the treatment Phase 1	COVID-19
27	NCT04615429	Hospital Universitario Puerta de Hierro-Majadahonda, Madrid, Spain	04/11/2020 08/02/2021	20 18 Years and older [Adult, Older Adult]	Biological: Mesenchymal stromal cells Administration of one single dose of allogenic Mesenchymal stromal cells Approximately 1 × 10^6^ MSC/kg Phase 2	COVID-19
28	NCT04625738	Central Hospital, Nancy, France	06/11/2020 06/08/2022	30 18 Years and older [Adult, Older Adult]	Ex vivo expanded Wharton’s Jelly derived mesenchymal stem cells will be infused at day 0, day 3 and day 5 [+/− 1 day], in patients with moderate to severe ARDS with a mechanical ventilation. Day 0: 1.10^6^ MSC/kg day 3: 0.5 × 10^6^ MSC/kg day 5: 0.5 x. 10^6^ MSC/kg Phase 2	COVID-19
29	NCT04629105	University of Maryland Medical Center Miami VA Healthcare System Miami, Florida, United States	16/11/2020 24/07/2021 20/07/2025	70 18 Years and older [Adult, Older Adult]	Biological: Longeveron Mesenchymal Stem Cells [LMSCs] Subjects with ARDS and acutely infected with SARS-CoV-2. Arm 1: 25 subjects treated with up to 3 doses of 100 million LMSCs Phase 1	COVID-19
30	NCT04657458	Direct Biologics, LLC Austin, Texas, United States	08/12/2020 31/12/2021	18 Years and older [Adult, Older Adult]	Bone Marrow Mesenchymal Stem Cell Derived Extracellular Vesicles Infusion Treatment Intravenous Infusion over 60 min Phase 2	COVID-19
31	NCT04728698	United States, California Fresno Community Hospital	28/01/2021 02/02/2021	100 18 Years and older [Adult, Older Adult]	Allogeneic culture-expanded adipose-derived mesenchymal stem cells [MSCs] 1 × 10^6^ MSCs/kg or 1.5 × 10^6^ MSCs/kg, depending on CRP level Phase 2	COVID-19
32	NCT04713878	University of Health Sciences Istanbul, Turkey	19/01/2021 20/05/2022	21 18 Years to 90 Years [Adult, Older Adult]	Intravenous Infusion of Mesenchymal stem cells A 8-Week Trial of Mesenchymal Stem Cells Therapy in Patients With COVID-19 Pneumonia	COVID-19
33	NCT04753476	Stem Cell and Cancer Research Indonesia Provincial Government of Central Java, Indonesia	15/02/2021 01/03/2022	48 Child, Adult, Older Adult	Injection of Hypoxic Secretome-MSCs intramuscular Day 1: 1 cc every 12 h, Day 2: 1 cc every 12 h, Day 3: 1 cc every 12 h. Patients will be given Standard treatment of Covid-19 which accordance with National protocol Phase 2	ARDS
34	NCT04780685	Saint John’s Health Center - Saint John’s Cancer Institute Santa Monica, California, United States	03/03/2021 31/12/2021	40 18 Years and older [Adult, Older Adult]	hMSCs will be administered intravenously Allogeneic mesenchymal bone marrow cells Phase 2	ARDS
35	NCT04798066	Hope Biosciences Stem Cell Research Foundation Sugar Land, Texas, United States	15/03/2021 20/03/2022	18 Years to 65 Years [Adult, Older Adult]	Biological: Autologous HB-adMSCs Autologous mesenchymal stem cells generated from adipose tissue	ARDS
36	NCT04798716	Mission Community Hospital Panorama City, California, United States	15/03/2021 15/12/2021	55 18 Years and older [Adult, Older Adult]	MSC-exosomes delivered intravenously every other day on an escalating dose: [2:4:8], [8:4:8], [8:8:8] Escalating dose 2 × 10^9^, 4 × 10^9^, 8 × 10^9^/Ml Phase 1/Phase 2	ARDS
37	NCT04898088	Istinye University, Istanbul, Turkey	21/05/2021 31/05/2022	30 18 Years to 65 Years [Adult, Older Adult]	Transplantation of Mesenchymal Stem Cells was performed at three 30-day intervals.	COVID-19
38	NCT04903327	Sorrento Therapeutics, Inc.	26/05/2021 03/06/2021 01/02/2022	100 18 Years and older [Adult, Older Adult]	A total of about 30 million cells from two vials of MSC will be infused intravenously into each subject on Days 0, 2, and 4. Allogeneic cell culture is what COVI-MSCs are. generated stem cells from adipose tissue Phase 2	ARDS

^a^NCT number: The National Clinical Trial number, ClinicalTrials.gov Identifier, https://clinicaltrials.gov

**Fig. 4:**
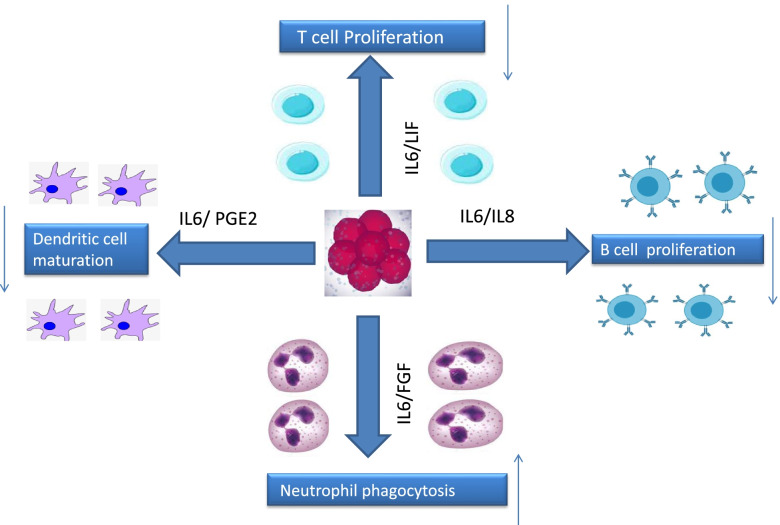
Effect of Mesenchymal stem cells (MSCs) on dendritic cell, B cell, T cell and Neutrophil. Mesenchymal stem cells with the effect of interleukin 6,8, LIF and PGE2 reduce cell secretion and increase the activity of dendritic cells

MSC-based treatments also showed encouraging results in the experimental therapy of lung failure by reducing alveolar collapse, collagen buildup, and cell death in the tissue. In addition, several researchers have achieved results. Patients with induced acute respiratory distress syndrome were treated for the first time with MSCs produced from allogeneic menstrual blood. They observed improved lung function after using MSCs [92]. The three preferred antiviral mechanisms that MSCs might exhibit in the context of a respiratory viral infection, such as COVID-19, include increased surface expressions of MSCs specific IFN-stimulated genes, secondary response to IFN, leading to the induction of MSC-stimulated genes that contribute to widespread viral resistance, and third modulation of the immune system. Aside from this, MSCs are ideal for treating SARS-COV-2 infection, which occurs when the immune and anti-inflammatory systems in the lungs get overwhelmed by cytokines (Fig. 4).

IFN-, an antiviral immune response, can activate MSCs and lead to the production of anti-inflammatory mediators, even if the mediators responsible for alleviating lung damage caused by the respiratory virus are still unclear [93–95]. Angiopoietin-1 and keratinocyte growth factors, released by MSCs, have been shown to play a role in regenerating alveolar-capillary barriers in acute respiratory syndrome. In addition, some literature has shown that the presence of a DAMP and PAMP mechanism causes lung inflammatory reactions. Due to their role in downstream cell signalling pathways, MSC activation is induced by Toll-like receptors (TLRs) triggered by viral RNA such as TLR3 in COVID-19 and viral non-methylated CpG-DNA such as TLR9. Stem cell treatments and secretory extracellular vesicles (EVs) have been found to be potential new medicines for reducing inflammation and repairing damaged tissue. Lung injury caused by COVID-19. Particular inhibitory miRNAs in EVs explain as mediating the protective effects of MSCs in pre-clinical models of viral acute lung injuries [65, 96, 97]. With the advent of COVID-19, one of the best strategies for treating or controlling the disease is to use antiviral drugs, targeting virus replication [98, 99]. These include favipiravir and remdesivir, which is an antiviral drug. However, because storm cytokines are found mainly in COVID-19, it is essential to consider drugs that inhibit the virus when replicating cytokine storms [100]. Because of this, MSC-Exos may be a suitable therapy for COVID-19. In some instances, using exosomes or secretions rather than mesenchymal stem cells themselves may be more effective and beneficial than traditional mesenchymal stem cell treatment [101]. With less toxicity and the ability to reach cells for more effective objectives through exosomes, exosomes can be extensively generated and prescribed [102]. In addition to its role in modulating the innate immune system, MSC-Exos can also be used as a drug delivery system. MSC-Exos can be modified in vivo to release exosomes that have the potential to modulate the immune system and can be cultured using different cytokines to show anti-inflammatory status [103]. Although MSC-Exos appear to be an appropriate therapeutic role for COVID-19, more clinical studies are needed to use them.

Additionally, it is critical to developing a strategy for preserving and isolating MSC-Exos for COVID-19 therapy. Furthermore, investigations are needed to understand COVID-19’s underlying mechanisms better so that MSC-Exo therapy may be improved for treatment. Additionally, determining the optimal dosage, delivery technique, and treatment strategy for MSC-Exos is critical. As a result of their more outstanding research in these areas than MSC-Exos, MSCs are primarily selected in COVID-19 clinical studies [104, 105].

### Severe COVID-19 and MSC-based therapy treatment options

**Fig. 5:**
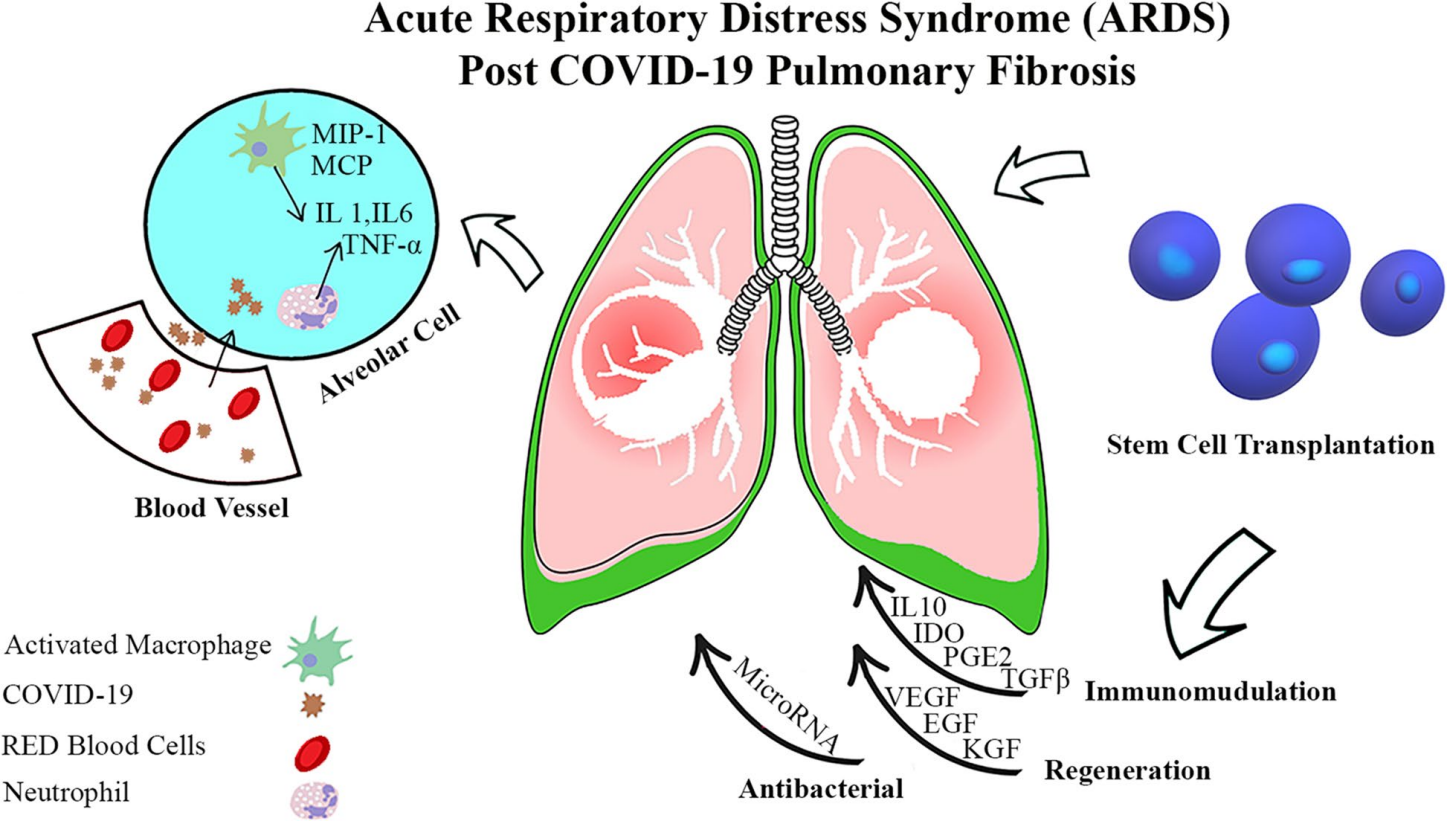
COVID-19 deficiency can lead to significant inflammatory-mediated consequences, such as ARDS. In these people’s lungs, immune cells such as macrophages and neutrophils are activated, pro-inflammatory cytokines [IL-6, TNF-] are produced, and endothelial cells are damaged. MSCs might be functional in this situation since they have immunomodulatory, regenerative and antibacterial effects

COVID-19 has a strong association with advancing years. Men over the age of 75 who have a history of diabetes, hypertension, heart disease, chronic respiratory disease, cancer, or a history of surgery have the highest mortality rate [106–108]. The cellular immune system must be used to combat COVID-19 virus [109, 110]. Italy has the highest elderly population in Europe and the world’s second-oldest population [111]. Italy also has one of the highest smoking rates, lung disease and ischemic heart disease [112]. Hence, the mortality rate in this country is higher compared to other countries [113]. However, young and healthy people can get the disease and die. The mortality rate from this virus is estimated at 4.3% [114–116]. Conventional treatments, such as hydroxychloroquine, are often not suitable and are not used in treatment. However, this drug is prescribed by some doctors. Recently, RamedSavier has been introduced as an antiviral drug [117]. Remdesivir is an adenosine analogue targeting viral RNA polymerase and inhibiting viral RNA synthesis [118]. Antiviral drug umifenovir, sold under the trade name Arbidol, is being studied and may be an alternative for treating the virus. COVID-19 [119–121]. Lopinavir/Ritonavir Neuraminidase, DNA inhibitors like Tenofovir Disoproxil or Lamivudine, and medicines like EK1 Peptide are alternatives to antiviral medications for COVID-19 therapy. Interferon recombinant has also been demonstrated to be effective [122–125]. Also, intravenous IVIG is prescribed for all ages. It can reduce inflammatory cytokines and increase effective anti-inflammatory mediators [126] prescribed of thymosin α can restitute the immune system of these people [127]. Plasmotherapy by injecting plasma into people recovering from Covid-19 due to the presence of specific IgM, IgG and IgA antibodies against COVID-19 antigen markers can neutralize the virus in patients, clear it and prevent it from replicating. So it helps patients recover faster [128–130]. Since most viral infections reach their peak in the first week, plasma therapy is more effective early in the illness [131–133]. As ACE2 receptor expression in the human kidney is approximately 100 times greater than in the lung, the kidney has become a primary target for COVID-19 infections. Therefore, blood purification helps to improve kidney function by reducing the activity of the kidneys. Blood purification also prevents cytokine storms by eliminating inflammatory and destructive factors [134–139]. In addition, blood purification balances electrolytes and the blood buffering system, resulting in homeostasis and increased oxygen loading capacity by red blood cells [140–142]. A novel strategy for treating this condition involves the use of mesenchymal stem cells [143]. According to clinical studies, patients with a severe disease state respond correctly to effective adjuvant therapy, which improves health and reduces mortality [144]. Current regenerative drug-based adjuvant treatment, including recovery plasma injections and mesenchymal stem cell transplants, has been admitted into clinics or clinical studies [145, 146]. The capacity of allogeneic mesenchymal stem cells and MSC-derived EVs to divide and proliferate may be proven as an effective adjuvant treatment with fast diffusion by intravenous injection of COVID-19. This therapy is now being evaluated and may become widely available soon [147, 148]. The first MSC injection into human patients began in 1993. Since then, in the last 25 years, MSC injections have been recorded in about 1000 clinical trials and treated more than 10,000 patients [149–151]. MSC has the properties of reducing the inflammatory response and strong repair [152]. Previously, mesenchymal stem cells were clinically evaluated to treat disorders associated with host-to-host transplantation [153], viral-induced immunological deficiencies, and chronic infection with human immunodeficiency virus, hepatitis B virus, and influenza virus [154, 155]. It has been shown that mesenchymal stem cells are attracted to the inflammatory location [156]. Inflammation follows the secretion of cytokines and attaches to the endothelium [157]. To summarize, the immune control mechanism of the MSC involves modifying immune activity and inhibiting immune cells implicated in inflammation that reach the lungs’ tissue [158–161]. These drugs also assist in the reduction of interferon IFN- production by natural killer cells (NK cells) and the alteration of the cytokine secretion profile by dendritic cells [162]. In various studies, mesenchymal stem cells in ALI and ARDS have been well described. MSCs perform their function by targeting infectious, inflammatory and endothelial agents. Mesenchymal stem cells have the ability to release KGF2, PGE2, IL-6, and IL-13 to aid in the process of phagocytosis [163–166]. In addition, lung injury from sources other than coronavirus has been the subject of several clinical research investigating the effect of mesenchymal stem cells [167]. Mesenchymal stem cells and their secreted secretions have immune, anti-inflammatory, anti-apoptotic functions (see Fig. 5) [99]. Recent studies have also shown anti-fibrotic properties in ALI and ARDS [168]. Macrophages’ M1 to M2 transition is facilitated by PGE2 and IL10, whereas IDO enhances the lungs’ antibacterial function [169, 170]. In addition, the cytokine activity of type B lymphocytes is inhibited by mesenchymal stem cells [153]. Mesenchymal stem cells help regenerate the capillary barrier, which protects alveolar cells through the action of growth and secretion factors [171, 172]. MSC-EV therapy was associated with immunoregulatory control in ALI models produced in mice [173]. The antibacterial effect of MSC is further demonstrated in inhibiting bacterial growth [174]. Numerous clinical trials have been conducted to evaluate the therapeutic benefits of MSCs and MSCs produced from MSCs in animal models of ALI, ARDS, and other lung inflammatory diseases [175, 176]. These results demonstrated a substantial decrease in inflammatory responses, increased oedema secretion, and epithelial damage regeneration [177].

According to a pre-clinical investigation, intravenous Infusion of mesenchymal stem cells increased access to the alveolar epithelium and pulmonary endothelium [178, 179]. One of the most lethal outcomes of SARS-COV-2 infection is an excessive immunological response, which, coupled with cytokine storms and acute respiratory distress syndrome, results in the failure and death of various bodily tissues, including the lungs [180–182]. Following intravenous injection, mesenchymal stem cells are confined in the injured lung and work directly with it to balance the immune system [183, 184]. The use of these cells can be effective in initiating a cytokine storm [185]. A peripheral or central vein is used to inject the cell. In some cases, cells can enter through the trachea [186]. Therefore, it can be considered that intratracheal administration may work better in Covid-19 patients [187]. However, all clinical trials to date have employed the intravenous injection of MSCs (Table 1), and the feasibility and efficacy of MSC delivery via intratracheal/bronchial injection remain unclear [188, 189]. Mesenchymal stem cells have been shown in pre-clinical investigations to be effective in the treatment of lung damage and ARDS [190]. MSC treatment has been proven to lower inflammatory cytokines such as IL-1, IL-1, IL-6, IFN-, and TNF- It becomes and boosts anti-inflammatory cytokines such as IL-4. However, in-vivo models have not yet revealed the specific mechanism of action. By raising alveolar air volume and decreasing alveolar thickness and inflammatory indicators, IL-5 and IL-10 help heal lung epithelial cell injury and improve alveolar fluid secretion. Since MSCs can contribute to enhanced performance, it must be stated [191–196]. If the results of preliminary investigations are verified in larger studies, mesenchymal stem cells may have a role in the therapy of illness by using derivatives of MSCs, such as medium or extracellular vesicles [197, 198].

## Conclusion

Various treatments have been introduced to treat COVID-19 disease. They are using mesenchymal cells, one of the treatments method that has shown promising results in clinical trials. As a consequence of the broad outbreak of the COVID-19 sickness and the widespread usage of numerous drugs, this disease has been successfully treated in a variety of destinations. So far, the use of these drugs has not shown promising results, and the disease is currently causing many deaths so that in some countries, the resulting mortality rate is higher than 6%. MSCs are one of the cells that modulate the immune system and, according to the mechanism described in this study, can play a critical role in the treatment of this fatal disease. Because researchers use immunosuppressive drugs (Tocilizumab) to alleviate COVID-19 symptoms in patients, these cells, as the mechanism of action, can significantly improve critical conditions. Using these cells has been reported in some viral diseases. The results of using these cells in viral diseases such as influenza have also been brilliant. Numerous studies are being conducted on these cells and their therapeutic role against COVID-19. This research has even progressed to phase 3. So far, the results have been satisfactory. It is hoped that these relevant results will be conclusive. Although the use of MSCs has shown positive effects, more research is needed to make widespread use of these.
